# JNK1 Mediates Lipopolysaccharide-Induced CD14 and SR-AI Expression and Macrophage Foam Cell Formation

**DOI:** 10.3389/fphys.2017.01075

**Published:** 2018-01-05

**Authors:** Dong An, Feng Hao, Chen Hu, Wei Kong, Xuemin Xu, Mei-Zhen Cui

**Affiliations:** ^1^School of Life Sciences, Jilin University, Changchun, China; ^2^Department of Biomedical and Diagnostic Sciences, College of Veterinary Medicine, University of Tennessee, Knoxville, TN, United States

**Keywords:** vascular biology, macrophage, lipopolysaccharide, signal transduction, CD14

## Abstract

Foam cell formation is the key process in the development of atherosclerosis. The uptake of oxidized low-density lipoprotein (oxLDL) converts macrophages into foam cells. We recently reported that lipopolysaccharide (LPS)-induced foam cell formation is regulated by CD14 and scavenger receptor AI (SR-AI). In this study, we employed pharmaceutical and gene knockdown approaches to determine the upstream molecular mediators, which control LPS-induced foam cell formation. Our results demonstrated that the specific c-Jun N-terminal kinase (JNK) pathway inhibitor, SP600125, but neither the specific inhibitor of extracellular signaling-regulated kinase (ERK) kinase MEK1/2, U0126, nor the specific inhibitor of p38 MAPK, SB203580, significantly blocks LPS-induced oxLDL uptake, suggesting that the JNK pathway is the upstream mediator of LPS-induced oxLDL uptake/foam cell formation. To address whether JNK pathway mediates LPS-induced oxLDL uptake is due to JNK pathway-regulated CD14 and SR-AI expression, we assessed whether the pharmaceutical inhibitor of JNK influences LPS-induced expression of CD14 and SR-AI. Our results indicate that JNK pathway mediates LPS-induced CD14 and SR-AI expression. To conclusively address the isoform role of JNK family, we depleted JNK isoforms using the JNK isoform-specific siRNA. Our data showed that the depletion of JNK1, but not JNK2 blocked LPS-induced CD14/SR-AI expression and foam cell formation. Taken together, our results reveal for the first time that JNK1 is the key mediator of LPS-induced CD14 and SR-AI expression in macrophages, leading to LPS-induced oxLDL uptake/foam cell formation. We conclude that the novel JNK1/CD14/SR-AI pathway controls macrophage oxLDL uptake/foam cell formation.

## Introduction

Atherosclerosis is a leading disease, which causes death and disability in developed countries. The accumulation of cholesterol esters within artery cells, especially in macrophages, converting them into foam cells, is fundamental to the pathogenesis of atherosclerosis (Brown and Goldstein, 1983; Steinberg et al., 1989). In early fatty streak lesions, macrophages that are differentiated from monocytes become matured and gather low-density lipoprotein (LDL) and modified LDL, such as oxidized low-density lipoprotein (oxLDL). Uptake of oxLDL through scavenging receptors (SRs) leads to the formation of mature lipid-laden macrophages, which are called foam cells. SR-AI has been reported to be dominantly expressed in mouse macrophages and plays an important role in modified LDL uptake (Kzhyshkowska et al., 2012).

Lipopolysaccharide (LPS), is also referred as an endotoxin, which has numerous biological activities and is responsible for many pathological conditions caused by Gram-negative bacteria. As the highly inflammatory constituent of the outer membrane of Gram-negative bacteria, LPS induces macrophage foam cell formation *in vitro* (Howell et al., 2011; Morishita et al., 2013) and leads to the formation of aortic lesions *in vivo* (Li et al., 2002; Westerterp et al., 2007; Gitlin and Loftin, 2009). The binding of LPS to its co-receptor, CD14, activates inflammatory toll-like receptor pathways, initiating the release of pro-inflammatory cytokines into the vasculature (Wright et al., 1990; Dunzendorfer et al., 2004; Levy et al., 2009). It has been reported that the expression of CD14 is associated with thrombosis in carotid plaques (Hermansson et al., 2014). Our recent study revealed that CD14 is a key mediator in LPS-induced foam cell formation in macrophages (An et al., 2017). We further demonstrated that SR-AI is the downstream mediator of CD14 in regulating LPS-induced foam cell formation (An et al., 2017). In the current study, we aimed to understand the upstream pathway, leading to the LPS-induced oxLDL uptake by macrophages/foam cell formation.

Mitogen-activated protein (MAP) kinases have been implicated in the development of atherosclerosis (Muslin, 2008). Conventional MAPKs include the extracellular signal-regulated kinase 1 and 2 (Erk1/2 or p44/42), the c-Jun N-terminal kinases 1–3 (JNK1-3) and the p38 isoforms. In this study, we evaluated whether MAPK activity mediates LPS-induced oxLDL uptake in macrophages/foam cell formation and how MAPKs mediate LPS-induced foam cell formation. In particular, we determined whether activation of these kinases influences the expression of CD14 and SR-AI. Understanding the signaling pathways that control foam cell formation will significantly contribute to designing therapeutic strategies for curing atherosclerosis.

## Materials and methods

### Reagents

Lipopolysaccharide (LPS) was obtained from Sigma-Aldrich Co (St. Louis, MO). Antibodies against mouse CD14 and SR-AI were from R&D system (Minneapolis, MN). Antibodies against mouse Phospho-Erk1/2, Phospho-p38, Phospho-JNK, JNK1 and JNK2 were from Cell Signaling Technology (Danvers, MA). Antibody against mouse GAPDH was from EMD Millipore (St. Charles, MO). The MEK1/2-specific inhibitor U0126, the p38-specific inhibitor SB203580 and the JNK-specific inhibitor SP600125 were from Cayman Chemical (Ann Arbor, MI). The RNeasy kit, Non-silencing siRNA, and JNK1 and JNK2 siRNAs were from Qiagen (Gaithersburg, MD). Human LDL and Dil-labeled oxLDL was purchased from Biomedical Technologies (Stoughton, MA).

### Cell culture

Bone-marrow progenitor cells were harvested from the femur section of 8–10 weeks old C57B/6 mice. Total bone marrow cells were washed once with PBS, resuspended in LADMAC conditioned medium, and plated in tissue culture dishes. To produce the LADMAC-conditioned medium, the LADMAC cell line (ATCC, Manassas, VA) was grown to confluence in 75-cm^2^ flasks for 5 days, followed by harvesting and filtering these culture supernatants. DMEM supplemented with 10% FBS and 20% LADMAC supernatant was used as the conditioned medium to foster the growth and differentiation of bone marrow macrophages. The conditioned medium, prepared in a similar manner, has been used to support the growth of bone marrow-derived macrophages because the LADMAC cell line is a source of M-CSF (Sklar et al., 1985; Olivas et al., 1995). After 6 days in culture, 95% of bone-marrow progenitor cells were differentiated into macrophages (bone marrow-derived macrophages, BMDMs) (An et al., 2017).

### Western blot analysis

Cultured mouse BMDMs were rinsed with cold PBS and lysed in Western blot lysis buffer (50 mM Tris-HCl, pH 6.8, 8 M urea, 5% mercaptoethanol, 2% SDS, and protease/phosphatase inhibitors) with sonication for 30 s on ice. Cellular proteins were separated by 10% SDS-PAGE and transferred to an Immobilon-P PVDF transfer membrane (Billerica, MA). Membranes were then probed with the specific antibodies, and the specific protein bands were viewed using Luminata Forte Western HRP substrate (Billerica, MA).

### siRNA treatment

BMDMs were transfected with non-silencing or specific siRNA (Qiagen) for 48 h, using Lipofectamine RNAiMAX reagent (Thermo Fisher Scientific) following the instructions provided by the manufacturer. On day 3, cells were cultured in serum free medium for 24 h, followed by treatment either with or without LPS.

### LDL oxidation

Oxidized LDL was prepared by dialyzing native LDL against 10 μM CuSO_4_ in PBS at room temperature for 24 h. The oxidation was stopped, and the lipoprotein bound metal ion was removed by dialyzing against PBS containing 0.5 mM EDTA at 4°C overnight (Chai et al., 1996).

### Oil red O staining

BMDMs were cultured on microscope cover glasses in 12-well plates and starved for 24 h. After the LPS treatment, the cells were incubated with native LDL or oxidized LDL at 37°C for 3.5 h. After the completion of treatments, cells were rinsed once with PBS, fixed in 4% paraformaldehyde at 4°C for 30 min. Subsequently, the cells were rinsed once with PBS and then stained with Oil Red O solution (Sigma Aldrich) at room temperature for 10 min. After staining, the cells were rinsed two times with PBS then stained with hematoxylin (Sigma Aldrich) for 1 min. Afterward, they were rinsed two times with PBS and observed by light microscopy (Nikon Eclipse E600 microscope) (Xu et al., 2010).

### Analysis of Dil-oxLDL uptake

Cellular uptake of Dil-oxLDL was measured using both fluorescence microscopy and plate reader. BMDMs were cultured in 24-well plates and then starved for 24 h. After LPS treatment for 18 h, cells were incubated with Dil-labeled oxLDL (Biomedical Technologies, Stoughton, MA) for 3.5 h and then rinsed with cold PBS containing 5% BSA two times. For fluorescence microscopy, cells grown on cover glass slides were fixed in 4% ice-cold paraformaldehyde solution for 30 min. Then the cells were rinsed with cold PBS containing 5% BSA two times. Afterwards, the coverslips were mounted on slides with permanent aqueous mounting medium (Biogenex, Fremont, CA), and the labeled oxLDL were analyzed by fluorescence microscopy with a Nikon Eclipse E600 microscope. For plate reader quantification, cells were further rinsed with PBS for two more times. Cells were then detached from the culture plate with 0.5% Triton X-100 in PBS. The fluorescence value was measured by the Synergy HT plate reader (BioTek, Winooski, VT) at 530 nm of excitation wavelength and 590 nm of emission wavelength (Barlic et al., 2009; Park et al., 2012).

### Statistical analysis

Results are means ± S.E. Comparisons between multiple groups were performed using one-way analysis of variance with *post-hoc t*-tests. Single comparisons were made using two-tailed, unpaired Student *t*-tests. A *p*-value of 0.05 was considered statistically significant.

## Results

### LPS activates ERK, p38 and JNK pathways in BMDMs

**Figure 1:**
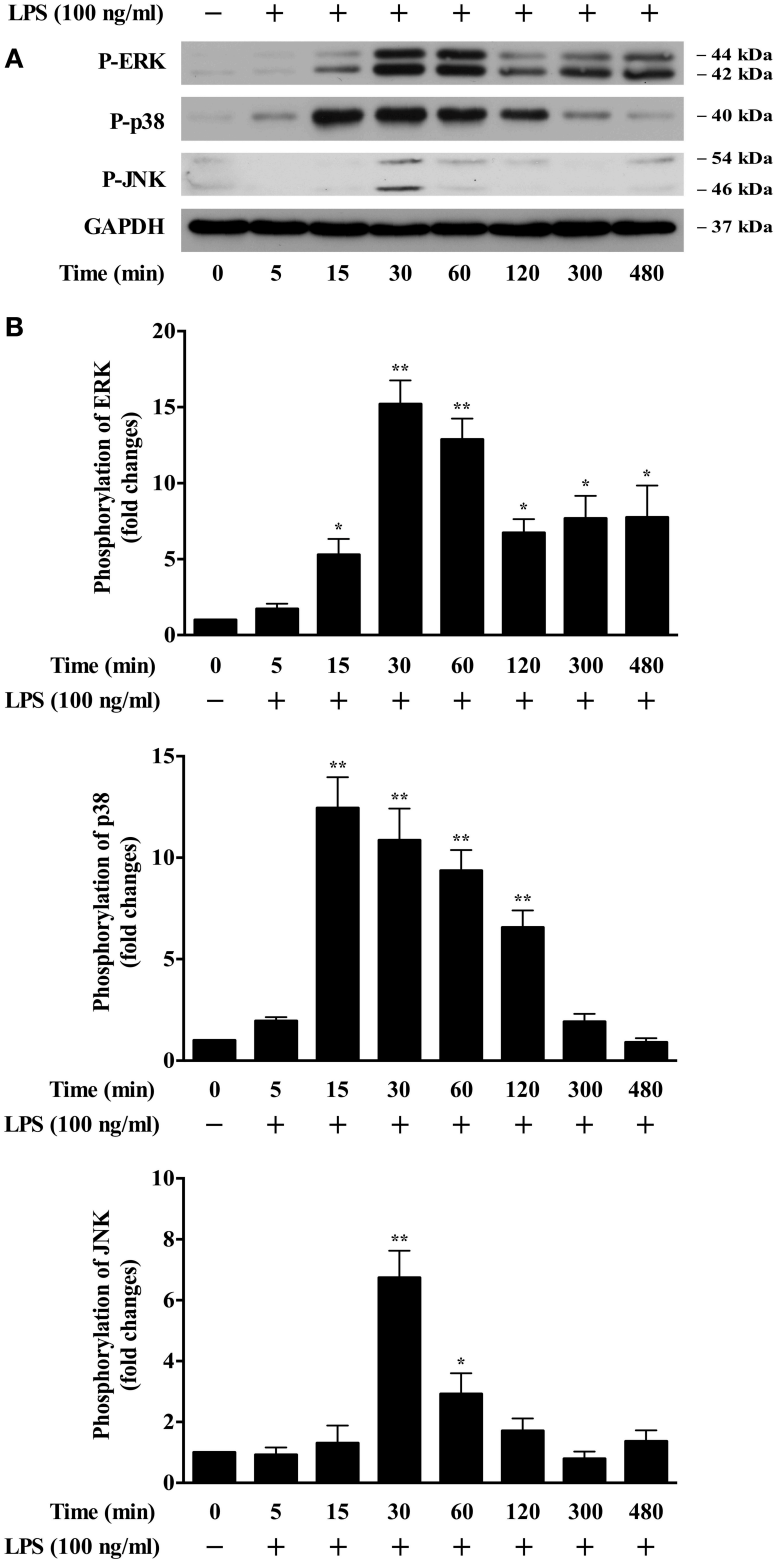
LPS induction of MAPK activation in BMDMs. **(A)** Western blotting data showed time courses of LPS induction of phosphorylation of ERK, p38, and JNK in BMDMs. Cultured BMDMs were starved for 24 h prior to LPS (100 ng/ml) stimulation for various times. Cell lysates were analyzed by Western blotting, using specific antibodies against p-ERK, p-p38, and p-JNK. GAPDH served as the loading control. The representative Western blot results shown were from three independent experiments. **(B)** results of the Western blot analysis were quantified as the densitometry value analyzed by UN-SCAN-IT gel 6.1 software. ^*^p < 0.05; ^**^p < 0.01 vs. control.

To pursue the upstream molecular determinants that control LPS-induced foam cell formation, we examined the possible signaling pathways involved. It has been demonstrated that the MAPKs signaling cascades play important roles in the development of atherosclerosis (Muslin, 2008). Whether these pathways are involved in LPS-induced oxLDL uptake/foam cell formation has been currently unrevealed. We isolated mouse bone marrow cells and then differentiated them into macrophages. We observed that the addition of LPS to the BMDMs highly and time dependently induced the phosphorylation of ERK, p38 and JNK MAPKs (Figures 1A,B). The activation peak for ERK is around 30–60 min; the activation peak for p38 is around 15–60 min and JNK at 30 min. These data suggest that the MAPK pathways might be involved in mediating LPS-induced oxLDL uptake by BMDMs.

### JNK pathway is involved in LPS-induced oxLDL uptake in BMDMs

**Figure 2:**
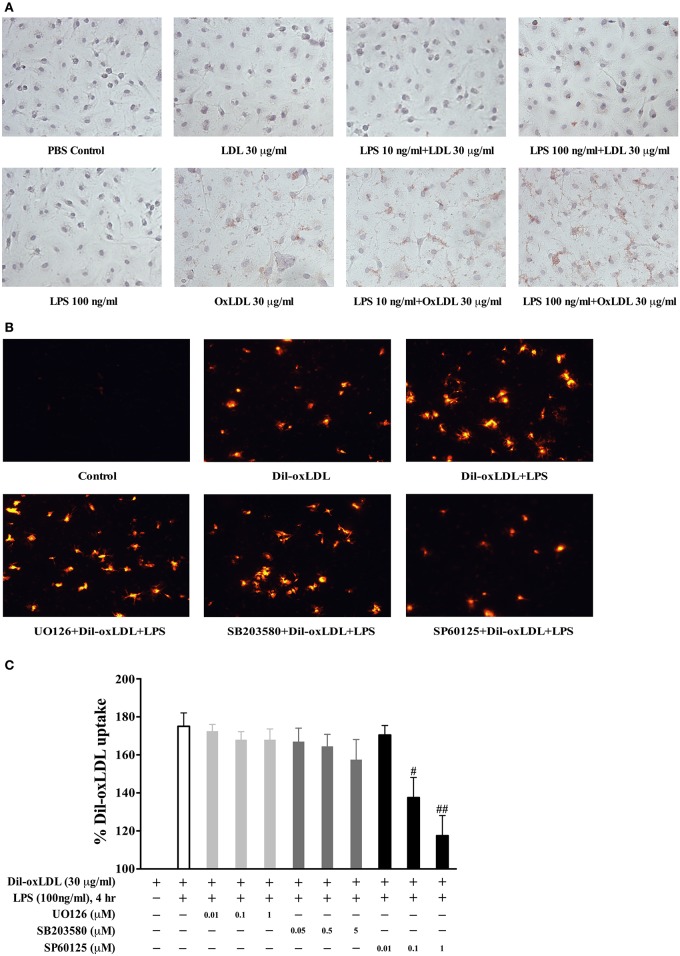
The involvement of JNK pathway in LPS induction of foam cell formation. **(A)** LPS effects on the uptake of oxLDL and LDL were examined by the Oil Red O staining with Nikon Eclipse E600 microscopy. BMDMs were stimulated with LPS for 18 h prior to the treatment of oxLDL or LDL for 3.5 h. **(B)** fluorescence microscopy results showed that pretreatment with the specific JNK inhibitor SP600125, but not the specific MEK inhibitor U0126 or p38 inhibitor SB203580, significantly blocked LPS-induced Di-oxLDL uptake. Twenty-four-hour serum free starved BMDMs were pretreated with various MAPK inhibitors (SP600125, 1 μM; SB203580, 5 μM; U0126, 1 μM) for 45 min prior to LPS treatment, followed by the addition of 30 μg/ml DiI-oxLDL for 3.5 h. The results were examined by fluorescence microscopy (Nikon Eclipse E600 microscopy); the Dil-oxLDL signals were shown in red. **(C)** pretreatment with specific JNK inhibitor SP600125, but not the specific MEK inhibitor U0126 or p38 inhibitor SB203580, dose-dependently blocked LPS-induced Dil-oxLDL uptake. The uptake of Dil-oxLDL fluorescence value was measured in Synergy HT plate reader at 530 nm (excitation) and 590 nm (emission). The Y axis represents the increased oxLDL uptake levels by LPS compared to the basal oxLDL uptake (uptake of oxLDL alone was considered as 100%). Quantified results were from three independent experiments. ^#^*p* < 0.05; ^##^*p* < 0.01 vs. LPS alone group.

The experimental results of the Oil Red O staining demonstrated that LPS dose dependently increased the uptake of oxLDL but not native LDL (Figure 2A). To determine whether these MAPK pathways contribute to LPS-induced oxLDL uptake in BMDMs, we examined the influence of MAPK inhibitors on LPS-induced oxLDL uptake. As shown in Figure 2B, the visualized fluorescence microscopy data reveal that the specific JNK inhibitor, SP600125 (1 μM), nearly completely blocked LPS-induced oxLDL uptake in BMDMs. However, pretreatment of either the specific MEK1/2 inhibitor, U0126 or the specific p38 inhibitor, SB203580, had no noticeable effect on LPS-induced oxLDL uptake. To confirm the role of JNK in LPS-induced oxLDL uptake, we quantified the Dil-oxLDL uptake by using a fluorescence microplate reader. Fluorescence at 530 nm of excitation wavelength and 590 nm of emission wavelength was assessed. As shown in Figure 2C, the JNK inhibitor SP600125 dose-dependently inhibited LPS-induced oxLDL uptake, but neither the specific MEK1/2, U0126 nor the p38 inhibitor, SB203580, in all of these doses tested, had an effect on LPS-induced oxLDL uptake. These doses have been previously demonstrated dose-dependently blocking the activation of ERK and p38 (Cuenda et al., 1995; Favata et al., 1998). These data suggest a regulatory role of JNK in LPS-induced oxLDL uptake.

### JNK pathway mediates the LPS-induced expression of CD14 and SR-AI in BMDMs

**Figure 3:**
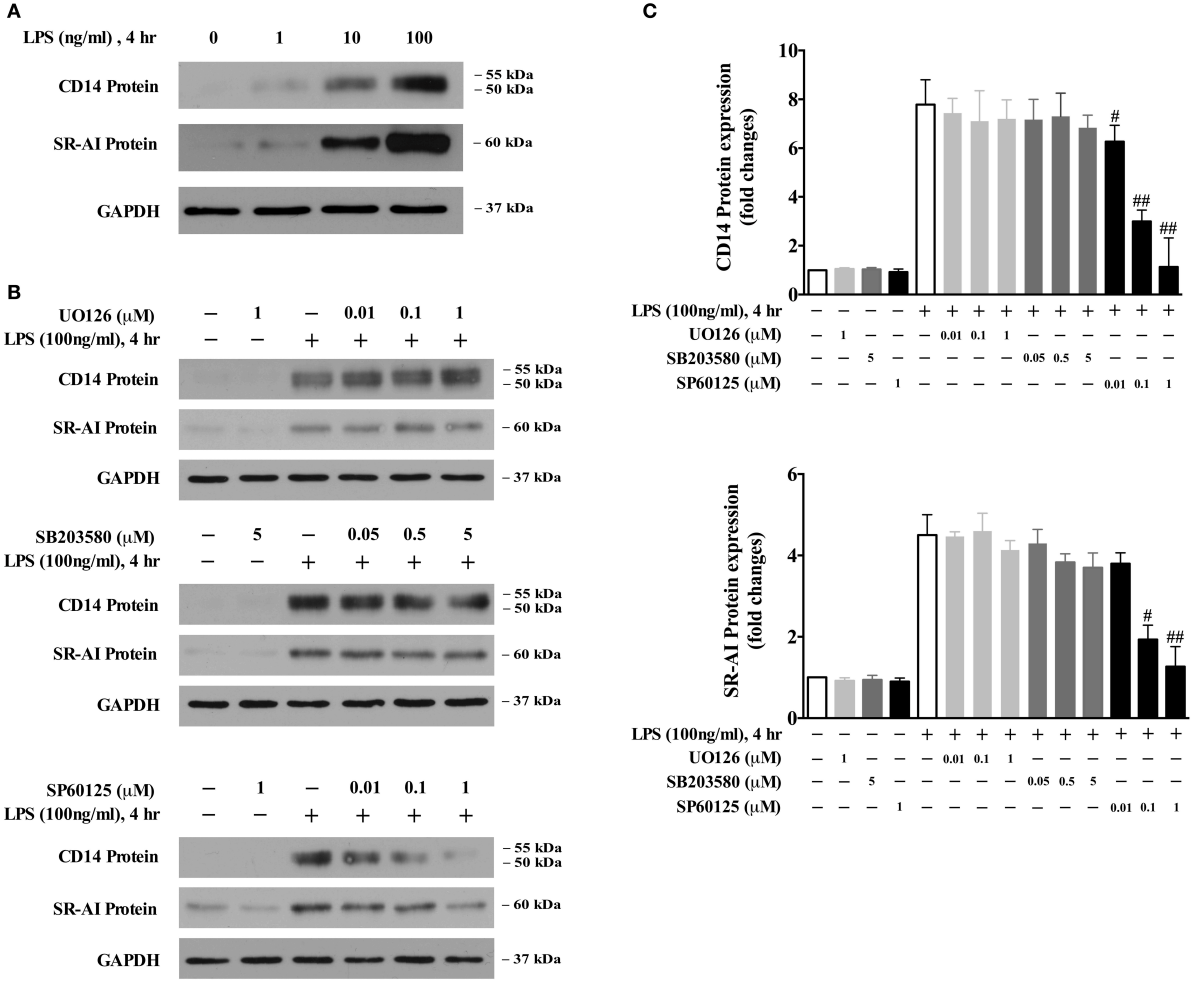
The JNK pathway contributes to CD14 and SR-AI expression. **(A)** Western blotting data showed that LPS dose-dependently induces CD14 and SR-AI expression in BMDMs. **(B)** Pretreatment with the specific JNK inhibitor SP600125, but not the specific MEK inhibitor U0126 or p38 inhibitor SB203580, dose-dependently blocked LPS-induced CD14 and SR-AI expression. Twenty-four-hour serum starved BMDMs were pretreated with various inhibitors for 45 min, and then 100 ng/ml LPS was added for 4 h. CD14 and SR-AI protein levels were determined by Western blotting. Data shown were from three independent experiments. **(C)** results of the Western blot analysis were quantified as the densitometry value analyzed by UN-SCAN-IT gel 6.1 software. ^#^*p* < 0.05; ^##^*p* < 0.01 vs. LPS alone group.

We recently identified that the CD14 and SR-AI axis mediates LPS-induced oxLDL uptake/foam cell formation (An et al., 2017). Our results demonstrated that LPS dose-dependently induced CD14 and SR-AI protein expression (Figure 3A). To identify the specific MAPK that mediates the expression of CD14 and SR-AI, we evaluated the influences of various MAPK inhibitors on the expression of CD14 and SR-AI. As shown in Figures 3B,C, the specific JNK inhibitor, SP60125, dose-dependently blocked LPS-induced expression of CD14 and SR-AI. However, neither the MEK specific inhibitor U0126 nor the p38 specific inhibitor SB203580 has a significant effect on the expression of CD14 and SR-AI. These data suggest that the novel JNK-CD14-SR-AI pathway mediates LPS-induced oxLDL uptake/foam cell formation.

### JNK1, but not JNK2, mediates LPS-induced expression of CD14 and SR-AI, which leads to oxLDL uptake in BMDMs

**Figure 4:**
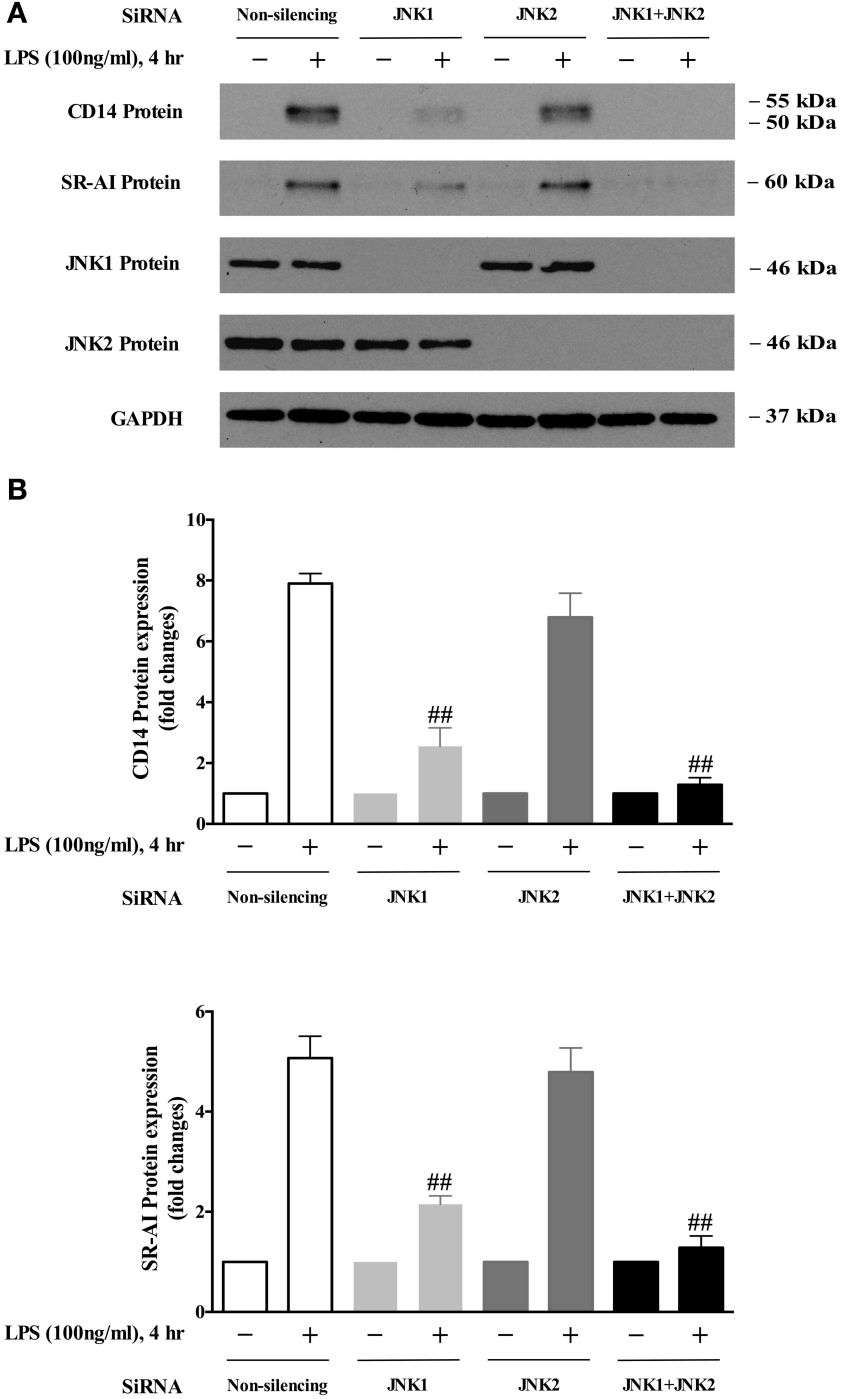
JNK 1 is the key mediator of LPS-induced CD14 and SR-AI expression. **(A)** Western blotting data showed that knockdown of JNK1 expression, but not JNK2, blocked LPS-induced CD14 and SR-AI expression in BMDMs. GAPDH served as the loading control. The representative Western blot results shown were from three independent experiments. **(B)** results of the Western blot analysis were quantified as the densitometry value analyzed by UN-SCAN-IT gel 6.1 software. ^##^*p* < 0.01 vs. Non-silencing group.

The JNK family consists of ten isoforms derived from three genes: JNK1, JNK2, and JNK3 (Gupta et al., 1996). It has been shown that JNK1 and JNK2 are widely expressed in various tissues; however, JNK3 is mainly expressed in the nervous system (Martin et al., 1996). To identify which JNK isoform is responsible for LPS-induced CD14 and SR-AI expression, specific siRNA of JNK1 or JNK2 were applied in BMDMs. As shown in Figures 4A,B, depletion of JNK1 largely abolished LPS-induced CD14 and SR-AI expression; however, knockdown of JNK2 with the specific siRNA of JNK2, has no significant effect on the expression of CD14 or SR-AI induced by LPS. Depletion of both JNK1 and JNK2 almost completely blocked LPS-induced expression of CD14 and SR-AI. These results demonstrate for the first time that JNK1 is required for LPS-induced CD14 and SR-AI expression.

### JNK1 mediates LPS-induced oxLDL uptake

**Figure 5:**
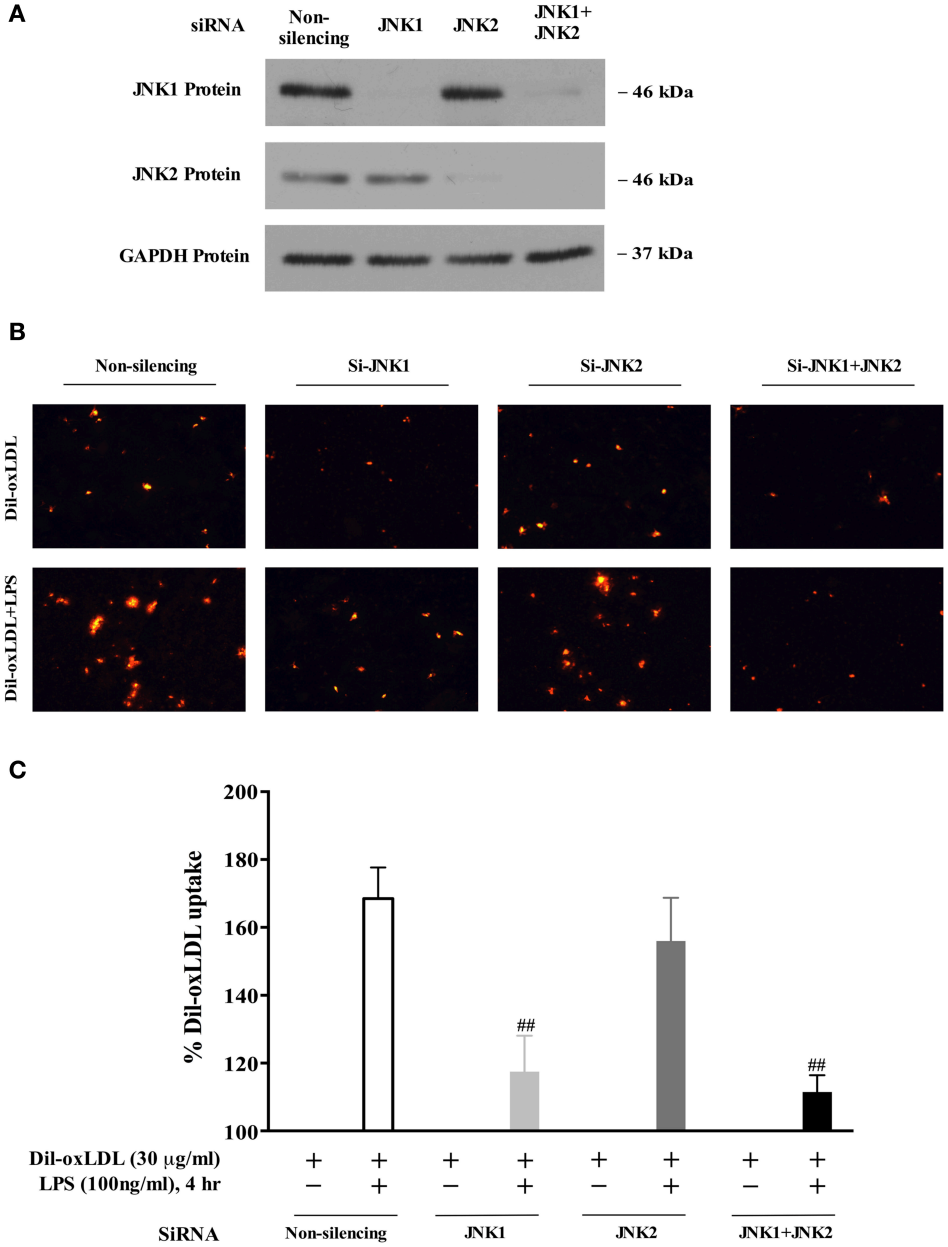
The JNK1 activity is required for LPS-induced oxLDL uptake in macrophages/foam cell formation. **(A)** knockdown efficiency of JNK1 and JNK2 with specific siRNAs in BMDMs was assessed in a Western blot analysis. **(B)** Visualized fluorescence microscopy data showed that knockdown of expression of JNK1 with specific siRNA blocked LPS-induced Dil-oxLDL uptake in BMDMs. The results were examined by fluorescence microscopy (Nikon Eclipse E600 microscopy); the Dil-oxLDL signals were shown in red. **(C)** quantitative fluorescence data indicated that knockdown of JNK1 expression using the specific JNK1 siRNA blocked LPS-induced Dil-oxLDL uptake in BMDMs. The quantitative analyses of oxLDL uptake using a fluorescence microplate reader were described in Figure 2. The Y axis represents the increased oxLDL uptake levels by LPS compared to the basal oxLDL uptake (uptake of oxLDL alone was considered as 100%). Quantified results were from three independent experiments. ^##^*p* < 0.01 vs. Non-silencing group.

We next examined whether JNK1 is required for LPS induction of oxLDL uptake. As shown in Figure 5A, the expression of JNK1 or JNK2 was successfully depleted using the specific JNK1 or JNK2 siRNA. We observed that the depletion of JNK1 blocked LPS-induced oxLDL uptake, whereas the depletion of JNK2 had no significant effect on oxLDL uptake (microscopy analysis, Figure 5B), indicating a key role of JNK1 in oxLDL uptake in macrophages. We substantiated our observation by quantifying the Dil-oxLDL uptake in a fluorescence microplate reader as described in Figure 2B. Our data show that depletion of JNK1 but not JNK2 nearly completely blocked oxLDL uptake (Figure 5C). Together, the results indicate that the specific isoform JNK1, but not JNK2 is required for LPS-induced oxLDL uptake in BMDMs.

## Discussion

Atherosclerosis is a chronic inflammatory disease, in which the formation of foam cells from macrophages is a crucial event, leading to the thrombotic complications. The unrestricted uptake of lipoproteins through scavenger receptor pathways, which is not limited by intracellular cholesterol levels, eventually leads to the formation of foam cells, the initial step in atherosclerosis (de Winther et al., 2000; Lusis, 2000). LPS, usually found in the outer membrane of Gram-negative bacteria, is a potential mediator of inflammatory responses. *Chlamydiae*-derived chlamydial LPS has been detected in atherosclerotic lesions (Hu et al., 1999). Repeated intravenous and intraperitoneal administration of LPS accelerates atherosclerosis in rabbits and *apoe*-/- mice (Lehr et al., 2001; Ostos et al., 2002; Engelmann et al., 2004; Westerterp et al., 2007). It has been shown that common chronic infection plays an important role in human atherogenesis (Kiechl et al., 2001). In a recent study, we reported that LPS via CD14 and SR-AI mediates oxLDL uptake in macrophages/foam cell formation (An et al., 2017). Our data also demonstrated that lysophosphatidic acid (LPA) synergistically enhances LPS-induced macrophage foam cell formation (An et al., 2017). LPA highly accumulates in human atherosclerotic lesion (Siess et al., 1999). A long-term, high-fat diet elevates LPA levels in rabbit plasma/serum (Tokumura et al., 2002) and in small intestines in atherosclerotic mice (Navab et al., 2015). Therefore, LPA may worsen atherosclerosis via enhancing macrophage foam cell formation. On the other hand, a recent *in vitro* evidence has shown that LPS enhances LPA secretion and autotaxin levels, which converts lysophosphatidylcholine to LPA (Awada et al., 2014), suggesting that LPS may trigger to increase LPA levels *in vivo*. Therefore, it is possible that LPS may deteriorate atherosclerosis via an increase of LPA secretion in atherosclerotic lesions.

In the current study, we identified that JNK pathway mediates LPS-induced oxLDL uptake via JNK cascade-triggered upregulation of CD14 and SR-AI. We further report that the JNK1 MAPK, but not JNK2 MAPK, is the key mediator for LPS-induced CD14 and SR-AI expression, which leads to the oxLDL uptake in BMDMs. This conclusion was drawn based on the following lines of evidence: (1) pretreatment of the specific JNK MAPK inhibitor SP600125 blocked LPS-induced oxLDL uptake/foam cell formation, (2) pretreatment of the specific JNK MAPK inhibitor SP600125 blocked LPS-induced CD14 and SR-AI expression and (3) depletion of the specific isoform JNK1, but not JNK2 abolished LPS-induced CD14 and SR-AI expression and LPS-induced oxLDL uptake/foam cell formation. Therefore, our data reveal that the novel JNK1/CD14/SR-AI pathway contributes to macrophage oxLDL uptake/foam cell formation.

Besides the recognition of bacterial LPS function in the innate immune system, which leads to uncontrollable cytokine production and results in fatal sepsis syndrome in humans (Triantafilou and Triantafilou, 2002), LPS-triggers oxLDL uptake in macrophages causing foam cell formation (Howell et al., 2011; Morishita et al., 2013). LPS has also been reported to be involved in cardiovascular disease via activated proinflammatory pathways (Mehta et al., 1998; Becker et al., 2001). LPS induces expression of inflammatory cytokines IL-6, MCP-1 and tumor necrosis factor-α (TNF-α) (Jatta et al., 2005; Yin et al., 2013). LPS induction of inflammatory cytokine expression is mediated by the NF-κB pathway, which contributes to intima hyperplasia (Vink et al., 2002; He et al., 2017). In addition, LPS induces tissue factor expression, contributing to thrombosis (Fei et al., 1993; Brand et al., 1994; Nakagomi et al., 2000). Therefore, LPS is an important inflammatory trigger for various inflammatory diseases.

In this study, we identified that the specific isoform JNK1 is required for mediating LPS-induced CD14 and SR-AI expression and foam cell formation. These data also indicate that JNK pathway is essential for the development of atherosclerosis. This conclusion is supported by previous studies, which demonstrated that inhibition of JNK pathway, but not the ERK pathway abolished oxLDL-induced foam cell formation (Rahaman et al., 2006) and that inhibition of JNK activation attenuated low shear stress-induced atherosclerosis in ApoE-deficient mice (Wang et al., 2011).

LPS-induced JNK1 activation may contribute to CD14 and SR-AI expression via transcriptional regulation. It has been shown that once JNKs have been activated, these kinases translocate to the nucleus (Cavigelli et al., 1995), where they phosphorylate c-Jun and thereby trigger the target gene transcriptional activity (Karin, 1995). Jun and Fos are family members of transcription factor AP1 (activator protein-1) (Angel and Karin, 1991). It has been reported that AP-1 is critical for both CD14 and SR-AI expression (Mietus-Snyder et al., 1998; Iwahashi et al., 2000). Our data suggest that, JNK1 may, via the activation of AP1 family, activate the transcriptional machinery of CD14 and SR-AI genes to increase their expression, which in turn contributes to oxLDL uptake in macrophages/foam cell formation, resulting in atherosclerosis.

## Ethics statement

This study was carried out in accordance with the guidelines of the National Institutes of Health. The protocol was approved by the University of Tennessee Institutional Animal Care and Use Committee.

## Author contributions

M-ZC conceived the idea and coordinated the research. DA, FH, and CH designed and performed experiments and analyzed data. DA prepared the figures. DA, XX, WK, and M-ZC analyzed and interpreted data. DA and M-ZC wrote the manuscript. All authors edited, revised, and approved the final version of the manuscript.

### Conflict of interest statement

The authors declare that the research was conducted in the absence of any commercial or financial relationships that could be construed as a potential conflict of interest.
